# CircPLCE1 facilitates the malignant progression of colorectal cancer by repressing the SRSF2‐dependent PLCE1 pre‐RNA splicing

**DOI:** 10.1111/jcmm.16753

**Published:** 2021-06-26

**Authors:** Zhilei Chen, Hongyu Chen, Lei Yang, Xiangnan Li, Zhenjun Wang

**Affiliations:** ^1^ Department of General Surgery Beijing Chao‐Yang Hospital Capital Medical University Beijing China

**Keywords:** circPLCE1, colorectal cancer, RNA splicing, SRSF2

## Abstract

Studies have demonstrated that circular RNAs (circRNAs) play important roles in various types of cancer; however, the mechanisms of circRNAs located in the nucleus have rarely been explored. Here, we report a novel circular RNA circPLCE1 (hsa_circ_0019230) that facilitates the malignant progression of colorectal cancer (CRC) by repressing serine/arginine‐rich splicing factor 2 (SRSF2)‐dependent phospholipase C epsilon 1 (PLCE1) pre‐RNA splicing. Quantitative real‐time polymerase chain reaction was used to determine the expression of circPLCE1 in CRC tissues and cells. Cell Counting Kit‐8, Transwell and flow cytometric assays were used to assess the role of circPLE1 in CRC cell proliferation, migration and apoptosis, respectively. An animal study was conducted to test the role of circPLCE1 in vivo. Furthermore, catRAPID and RPISeq were used to predict the possible binding proteins of circPLCE1. RNA fractionation and RNA immunoprecipitation assays were used to confirm the RNA‐protein interaction. In this study, we found that circPLCE1 was more significantly down‐regulated in CRC tissues compared with that in adjacent normal tissues. However, circPLCE1 knockdown suppressed CRC cell proliferation, migration and invasion and increased apoptosis. Nude mouse experiments showed that ectopic expression of circPLCE1 dramatically increased tumour growth in vivo. Mechanistically, circPLCE1 directly bound to the SRSF2 protein, repressing SRSF2‐dependent PLCE1 pre‐RNA splicing, resulting in the progression of CRC. Individually mutating the binding sites of circPLCE1 abolished the inhibition of PLCE1 mRNA production. Our study revealed a novel molecular mechanism in the regulation of PLCE1 and suggested a new function of circular RNA.

## 1 INTRODUCTION

Phospholipase C epsilon 1 (PLCE1) is the most essential member of the phospholipase family.^1^ PLCE1 catalyses the hydrolysis of phosphatidylinositol 4,5‐bisphosphate to generate two secondary messengers inositol 1,4,5‐triphosphate and diacylglycerol.^2^ These products subsequently initiate a cascade of intracellular responses that result in cellular proliferation, differentiation and apoptosis.^3^ In addition, PLCE1 is a suppressor of P53 by promoting p53 promoter methylation.^4^ To date, many studies have reported that PLCE1 may play an essential role in carcinogenesis and progression of various human cancers, including oesophageal cancer,^5^ lung cancer,^6^ colorectal cancer (CRC)^7^ and hepatocellular carcinoma.^8^ However, little information has been reported regarding the regulation of PLCE1 protein in tumours.

Most genes in the human genome contain numerous exons interspersed with introns that undergo RNA splicing to form mature mRNA and protein products.^9^ Pathological abnormal splicing is a crucial component of cancer development and progression, affecting critical cancer‐associated gene expression.^10^ Serine/arginine‐rich splicing factor 2 (SRSF2) is an SR protein that consists of an RNA recognition motif (RRM) domain and an arginine‐serine (RS) domain.^11^ SRSF2 frequently binds to specific splicing enhancer sequences and functions as a general splicing activator.^12^ Remarkably, SRSF2 regulates transcriptional elongation in a gene sequence‐selective manner.^13^ To date, the role of SRSF2 in the formation of mature PLCE1 mRNA has not been reported.

Circular RNAs (circRNAs) are generated from back‐splicing of pre‐RNAs to form covalently closed loops.^14^ Most circRNAs arise from constitutive exons, whereas others contain introns. The biogenesis of circRNAs is abundant, stable, conserved and cell type‐specific, indicating that circRNAs have potential regulatory roles.^15^ Most current circRNA studies focus on circRNA as a sponge of miRNA molecules.^16^ Moreover, endonuclear circRNAs have been discovered to compete against classical pre‐RNA splicing, thereby reducing parent gene mature mRNA production.^17^, ^18^ Here, we provide the first evidence for a molecular mechanism by which circPLCE1 reduces the expression of PLCE1 mRNA by binding to SRSF2 protein in CRC. These results indicate that circPLCE1 is a potential therapeutic target for CRC.

## 2 MATERIALS AND METHODS

### 2.1 Colorectal cancer (CRC) tissues and cell lines

Fresh CRC tissues and matched adjacent normal tissues were acquired from 41 patients who underwent surgery between 2018 and 2019 at Beijing Chao‐Yang Hospital. All resected fresh tissues were immediately frozen in liquid nitrogen and stored at −80℃ until use. Written informed consent was signed by the patients or their families before the surgery began. The study was approved by the Ethics Committee of Beijing Chao‐Yang Hospital affiliated with the Capital Medical University.

Six CRC cell lines including SW480, SW620, HCT116, HT29, RKO and lovo were all purchased from ATCC. HCT116 cells were cultured in McCoy's 5A medium supplemented with 10% foetal bovine serum (FBS), and others were cultured in Dulbecco's modified Eagle medium supplemented with 10% FBS and then maintained in a humidified atmosphere of 5% CO_2_ at 37℃.

### 2.2 RNA extraction, reverse transcription and quantitative real‐time polymerase chain reaction analysis

TRIzol reagent (Invitrogen) was used for total RNA extraction from CRC cells and tissues according to the manufacturer's protocol. To determine the levels of circRNA, pre‐RNA and mRNA, total RNA was used for reverse transcription by using the PrimeScript RT Reagent Kit (Takara). Subsequently, quantitative real‐time polymerase chain reaction (qRT‐PCR) analysis was performed using TB Green Premix Ex Taq II (Takara). Reaction conditions were as follows: 95.0℃ 30 s, 1 cycle; 95.0℃ 5 s, 60.0℃ 34 s, 40 cycles. GAPDH was used for the normalization of circRNA, pre‐RNA and mRNA. The relative RNA expression levels were calculated using the 2^‐△△Ct^ method. The primer sequences are listed in Supplementary Table 1.

**TABLE 1:** The correlation between circPLCE1 expression and clinicopathological features in 41 CRC patients

Clinicopathological features	Total (n = 41)	circPLCE1 expression		*p* value
		High (%)	Low (%)	
Age (years)				
≥65	22	9 (40.9%)	13 (59.1%)	0.16
<65	19	12 (63.2%)	7 (36.8%)	
Gender				
Male	24	14 (58.3%)	10 (41.7%)	0.28
Female	17	7 (41.2%)	10 (58.8%)	
Tumour size (cm)				
≥5	23	13 (56.5%)	10 (43.5%)	0.44
<5	18	8 (44.4%)	10 (55.6%)	
Differentiation				
Poor	4	2 (50.0%)	2 (50.0%)	0.96
High	37	19 (51.4%)	18 (48.6%)	
Tumour site				
Rectum	11	5 (45.5%)	6 (54.5%)	0.65
Colon	30	16 (53.3%)	14 (46.7%)	
Depth of invasion				
T3/T4	37	21 (56.8%)	16 (43.2%)	0.03*
T1/T2	4	0 (0%)	4 (100%)	
Lymph node metastasis				
N1/N2	15	14 (93.3%)	1 (6.7%)	<0.001***
N0	26	7 (26.9%)	19 (73.1%)	
Distant metastasis				
M1	4	3 (75.0%)	1 (25.0%)	0.32
M0	37	18 (48.6%)	19 (51.4%)	
TNM tumour stage				
III+IV	15	14 (93.3%)	1 (6.7%)	<0.001***
I+II	26	7 (26.9%)	19 (73.1%)	
Lymphovascular invasion				
Present	22	13 (59.1%)	9 (40.9%)	0.28
Absent	19	8 (42.1%)	11 (57.9%)	

* *P* < .05, ***P < .001.

### 2.3 Small interfering RNAs and transfection

To silence circPLCE1, two antisense oligonucleotides (siRNAs) against the circPLCE1 junction site were designed and synthesized by GenePharma. The CRC cell lines SW480 and HCT116 were chosen for the transfection and subsequent functional tests. All siRNAs were transfected at a final concentration of 75 nM using Lipofectamine 3000 reagent (Invitrogen). All the siRNA sequences used are listed in Supplementary Table 2.

**TABLE 2:** The correlation between PLCE1 mRNA expression and clinicopathological features in 41 CRC patients

Clinicopathological features	Total (n = 41)	PLCE1 mRNA expression		*p* value
		High (%)	Low (%)	
Age (years)				
≥65	22	10 (45.5%)	12 (54.5%)	0.43
<65	19	11 (57.9%)	8 (42.1%)	
Gender				
Male	24	13 (54.2%)	11 (45.8%)	0.65
Female	17	8 (47.1%)	9 (52.9%)	
Tumour size (cm)				
≥5	23	10 (43.5%)	13 (56.5%)	0.26
<5	18	11 (61.1%)	7 (38.9%)	
Differentiation				
Poor	4	2 (50.0%)	2 (50.0%)	0.96
High	37	19 (51.4%)	18 (48.6%)	
Tumour site				
Rectum	11	5 (45.5%)	6 (54.5%)	0.65
Colon	30	16 (53.3%)	14 (46.7%)	
Depth of invasion				
T3/T4	37	19 (51.4%)	18 (48.6%)	0.96
T1/T2	4	2 (50%)	2 (50%)	
Lymph node metastasis				
N1/N2	15	4 (26.7%)	11 (73.3%)	0.02*
N0	26	17 (65.4%)	9 (34.6%)	
Distant metastasis				
M1	4	0 (0%)	4 (100%)	0.03*
M0	37	21 (56.8%)	16 (43.2%)	
TNM tumour stage				
III+IV	15	4 (26.7%)	11 (73.3%)	0.02*
I+II	26	17 (65.4%)	9 (34.6%)	
Lymphovascular invasion				
Present	22	8 (36.4%)	14 (63.6%)	0.04*
Absent	19	13 (68.4%)	6 (31.6%)	

* *P* < .05.

### 2.4 circPLCE1‐overexpressing lentivirus construction and infection

To overexpress cirPLCE1, full‐length human circPLCE1 cDNA was synthesized and cloned into the expression vector pHBLV‐CMV‐circ‐EF1‐Puro‐N by Hanbio. The control vector or circPLCE1‐overexpression vector was packaged into the lentivirus and infected into CRC cells. Puromycin was used to select stable circPLCE1‐overexpressing cells for at least 2 weeks after infection.

### 2.5 Cell proliferation and colony formation assays

Cell proliferation was detected using the Cell Counting Kit‐8 (CCK‐8) assays (Dojindo Laboratories). The treated cells were allowed to grow in 96‐well plates and incubated with 10 μL of CCK‐8 solution for 2 h at 37℃. The absorbance was measured at 450 nm using a microplate reader (Thermo Fisher Scientific). The proliferation rate was measured at 0, 24, 48, 72 and 96 h after transfection. All experiments were performed five times independently.

Colony formation assays were used to evaluate the clonogenic ability of the CRC cells. Furthermore, 1000 cells per well were seeded into 6‐well plates, and 14 days later, 4% paraformaldehyde was used to fix the cells, and crystal violet staining was used to visualize the clones. We counted the clones that were 2 mm or greater in size under a light microscope. The average number of colonies was determined from three independent experiments.

### 2.6 Transwell migration and invasion assay

Transwell assays were performed to evaluate the migration and invasion capabilities of CRC cells. For the migration assay, 200‐μL serum‐free medium containing 10^5^ CRC cells was added into the upper chambers of Transwells for 24 h. Similarly, for the invasion assay, 2 × 10^5^ CRC cells were added to Matrigel‐precoated Transwell upper chambers and incubated for 48 h. Medium containing 20% serum was added to the lower chambers as a chemoattractant. Eventually, the migrated and invaded cells were stained with crystal violet and counted using a Leica DM4000B microscope (Leica).

### 2.7 Flow cytometric assays of cell cycle and apoptosis

Cell cycle distribution and cell apoptosis rate were measured by flow cytometry. After treatment, CRC cells were collected and mixed with 75% ethanol at 4℃ overnight. The following day, the cells were labelled with PI/RNase Staining Buffer for 30 min before detection. The percentages of cells in different phases of the cell cycle were analysed using a FACSCalibur flow cytometer.

For the apoptosis assay, the cells were rinsed with phosphate‐buffered saline and resuspended in 500‐μL binding buffer. Fluorescein isothiocyanate and propidium iodide (KeyGen Biotech) were added and incubated for 15 min according to the manufacturer's instructions. Subsequently, the flow cytometer FACSCalibur was used to assess early cell apoptosis.

### 2.8 Tumour formation in vivo

Six‐week‐old male BALB/c nude mice were purchased from the Charles River Laboratories. For the tumour formation experiment, 1 × 10^6^ stable circPLCE1‐overexpresing HCT116 cells or NC cells were subcutaneously injected into either side of the back of each mouse (five in each group). Fifteen days later, tumours were harvested from mice. Tumour volume (mm^3^) was calculated as follows: 1/2 × length × width^2^. All animal experiments were conducted in compliance with the Animal Protection Law of the People's Republic of China‐2009 for experimental animals and were approved by the Ethics Committee of Animal Experiments of Beijing Chao‐Yang Hospital.

### 2.9 Western blot

After removing the culture supernatants, the cells were lysed using radioimmunoprecipitation assay buffer (Beyotime). Total protein was harvested and quantified using a BCA Reagent Kit (Beyotime). In brief, 30 μg of protein was separated by electrophoresis on a 10% sodium dodecyl sulphate‐polyacrylamide gel. Partitioned proteins were transferred onto polyvinylidene fluoride membranes, which were blocked in 5% non‐fat milk and then incubated with primary antibodies against PLCE1 (1:500, Biorbyt, orb335375) and β‐actin (1:5000, ProteinTech, 66009‐1‐lg) at 4℃ overnight. Horseradish peroxidase‐conjugated anti‐rabbit or anti‐mouse IgG antibody was used as the secondary antibody. Bands were detected using a chemiluminescence system (Beyotime).

### 2.10 Bioinformatic analysis

The secondary structure of circPLCE1 was formed by RNAfold, whereas the secondary structure of the SRSF2 protein was acquired by UniProt. We used catRAPID (http://service.tartaglialab.com/page/catrapid_group) to predict the target proteins of circPLCE1. In addition, the interaction strength was determined using the RPISeq program (http://pridb.gdcb.iastate.edu/RPISeq/index.html).

RNA‐binding residues in SRSF2 were predicted using RNABindRPlus and Pprint web tools. We used the protein‐RNA docking analysis tool NPDock server (http://genesilico.pl/NPDock) to discover the possible interaction of circPLCE1 with SRSF2 RRM.

### 2.11 Cytoplasmic and nuclear RNA fractionation

The subcellular localization of circPLCE1 was detected using a PARIS Kit according to the manufacturer's protocol (Ambion, AM1921). GAPDH mRNA and U6 served as controls.

### 2.12 RNA immunoprecipitation assay

An EZ‐Magna RIP Kit (Millipore) was used to determine the binding of RNA and proteins. Twenty million SW480 cells were lysed in an equal pellet volume of complete RIP lysis buffer on ice for 5 min, and the cell extract was incubated with magnetic beads conjugated with anti‐SRSF2 antibody (Abcam, ab204916) or IgG (PP64B) overnight at 4℃. Subsequently, the immunoprecipitates were digested with proteinase K at 55℃ for 30 min. Finally, the immunoprecipitated RNAs were purified using TRIzol and ethanol precipitation and subjected to qRT‐PCR analysis.

### 2.13 Nucleic acid electrophoresis

PCR products were verified by 2% agarose gel electrophoresis with Tris acetate ethylenediaminetetraacetic acid running buffer (120 V, 30 min). A 100 bp DNA Marker was used as a DNA marker. The bands were visualized under ultraviolet irradiation.

### 2.14 Statistical analyses

All the analyses were performed using Statistical Package for the Social Sciences version 23.0 (IBM). The data are presented as the mean ±standard deviation from at least three independent experiments. Statistical significance was determined using the chi‐squared test, Student's t test, Wilcoxon signed rank test and one‐way analysis of variance, as appropriate. The association between circPLCE1 and PLCE1 mRNA expression in CRC tissues was analysed using the Pearson correlation coefficient. Statistical significance was set at *P* < .05.

## 3 RESULTS

### 3.1 circPLCE1 was identified as a tumour promoter in CRC progression

**FIGURE 1:**
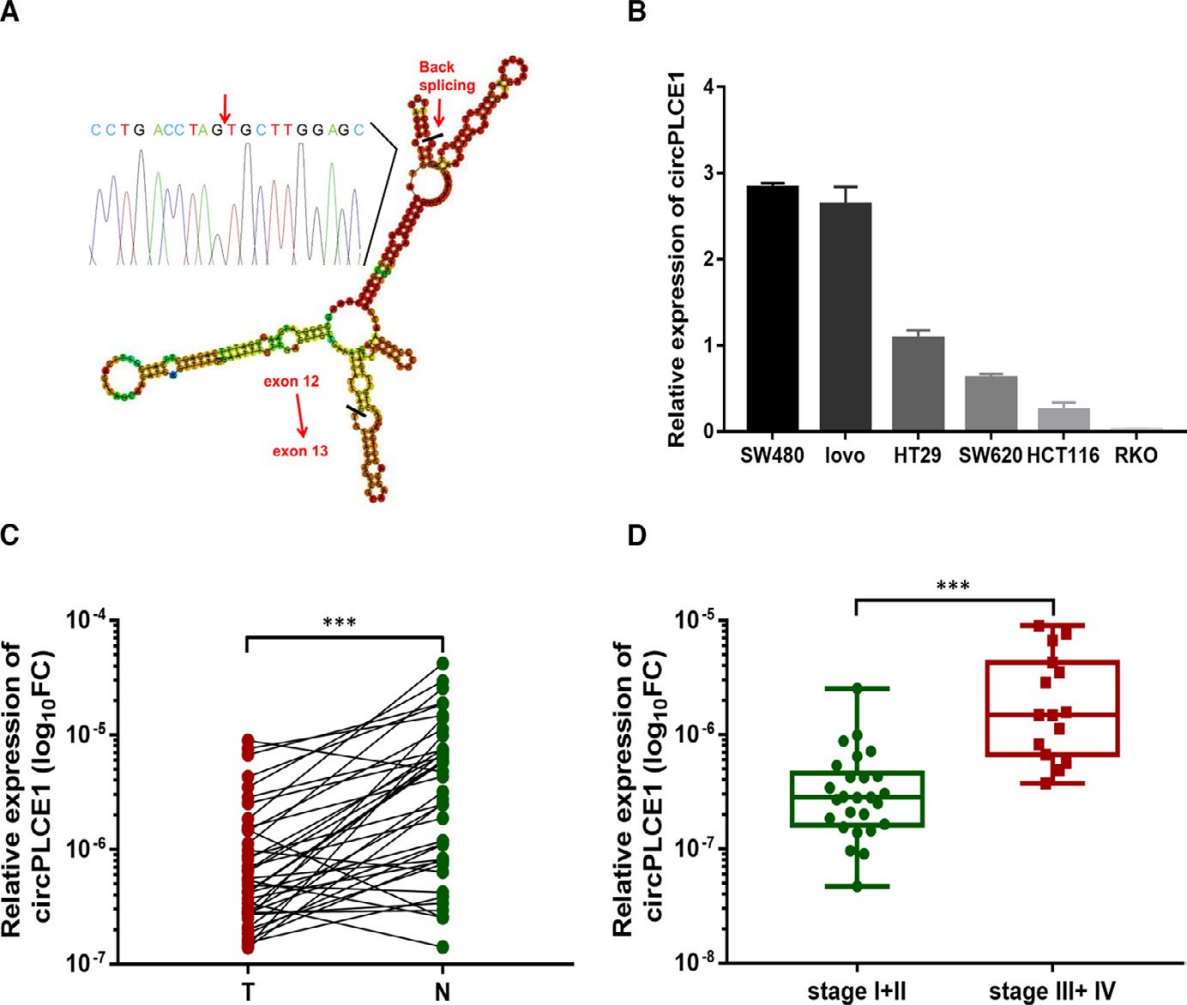
circPLCE1 validation and expression in colorectal cancer tissues and cells. (A) The secondary structure of circPLCE1 was formed by RNAfold. The presence of circPLCE1 was validated by qRT‐PCR and subsequent Sanger sequencing. The red arrow represents “head‐to‐tail” junction of circPLCE1. (B) Relative expression of circPLCE1 in CRC cell lines. (C) circPLCE1 was markedly down‐regulated in CRC tissues compared with paired adjacent mucosal tissues. (D) The circPLCE1 expression level in patients with different TNM stages. ***P <.001. T, tumour tissues; N, normal tissues

In our previous work, we analysed the expression levels of different circRNAs in CRC tissues relative to the paired adjacent mucosal tissues (four pairs).^19^, ^20^ We observed that the circular transcript of PLCE1 (circBase ID: hsa_circ_0019230) was decreased in tumour tissues. CircPLCE1 is derived from exons 12 and 13 of the PLCE1. Sequencing confirmed that the “head‐to‐tail” splice junction was identical to the circRNA database (Figure 1A). Next, we examined circPLCE1 levels in different CRC cell lines and found that circPLCE1 levels were highest in SW480 and lovo among other CRC cell lines (Figure 1B). To further investigate the correlation between circPLCE1 and CRC, we collected another 41 pairs of CRC tissues and adjacent normal mucosal tissues and performed qRT‐PCR to detect circPLCE1 expression. As shown in Figure 1C, CRC tissues expressed circPLCE1 at a notably lower level than the paired mucosal tissues. Interestingly, we observed a significant positive correlation between the patients’ tumour node metastasis (TNM) tumour stage and circPLCE1 expression level (*P* < .001, Figure 1D). Additionally, we also analysed the correlation between circPLCE1 expression and various clinicopathological characteristics of CRC patients (Table 1). The results indicated that the expression level of circPLCE1 was significantly associated with the depth of invasion (*P* = .03) and lymph node metastasis (*P* < .001). Taken together, circPLCE1 was identified as a tumour promoter in CRC progression.

### 3.2 circPLCE1 promotes CRC cell malignant phenotype in vitro and in vivo

**FIGURE 2:**
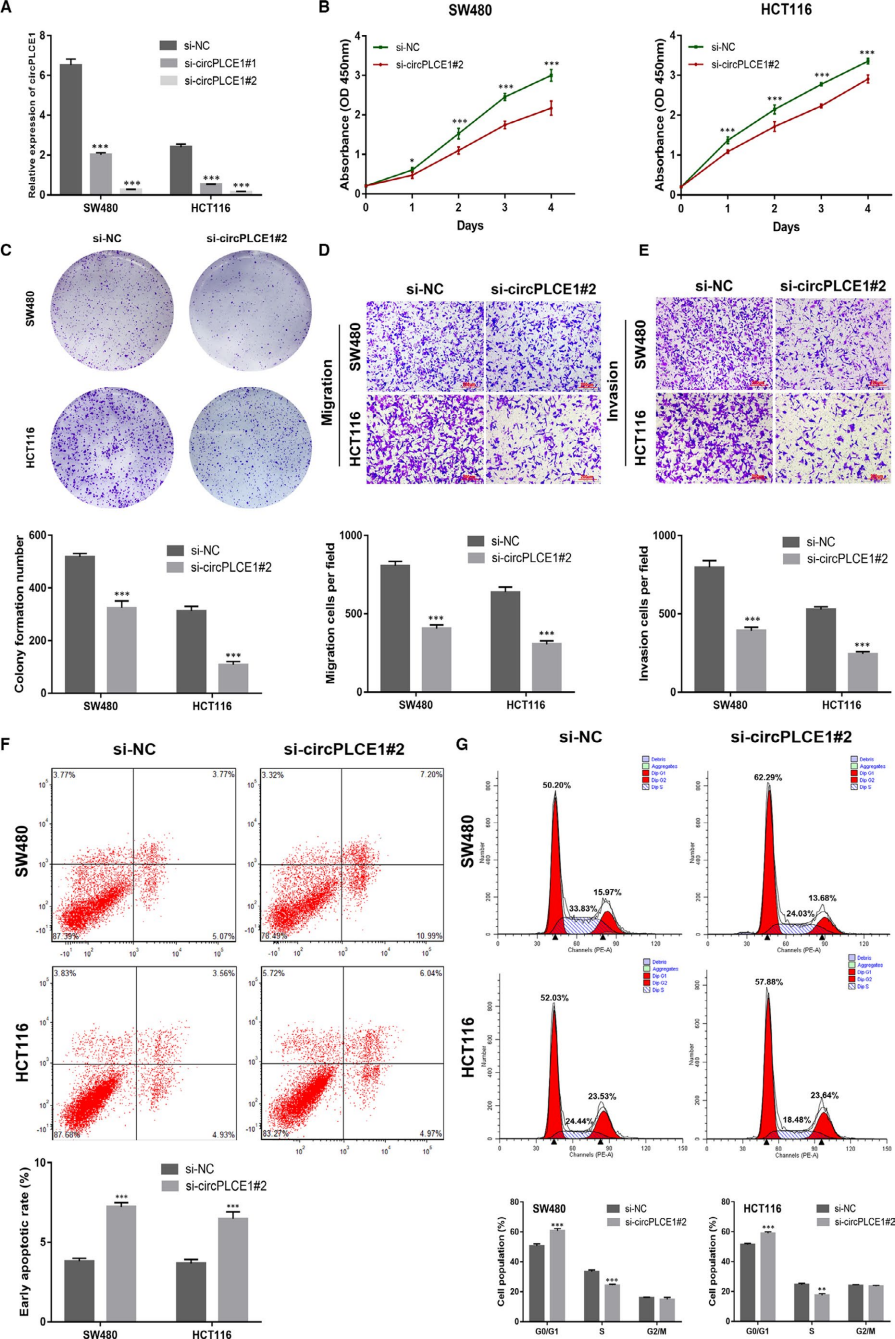
Knockdown of circPLCE1 inhibits cell proliferation, cell migration and cell cycle and promoted apoptosis. (A) Efficiency of siRNAs on circPLCE1 in SW480 and HCT116 detected by qRT‐PCR. (B) CCK‐8 assay was performed to determine cell proliferation in NC cells and si‐circPLCE1#2 cells. (C) Colony formation assays were conducted in differently treated cells. (D) Transwell migration assay in transfected SW480 and HCT116 cells. (E) Transwell invasion assay in transfected SW480 and HCT116 cells. (F) The cell cycle of SW480 and HCT116 cells after transfection. (G) Early apoptosis was assessed in cells transfected with NC or si‐circPLCE1#2 by flow cytometry. All data are presented as the means ±SD of at least three independent experiments. *P < .05, **P < .01, ***P < .001. Scale bar: 200 μm

SW480 and HCT116 cells were selected for subsequent experiments because of their significant differences in the expression of circPLCE1. To investigate the functional role of circPLCE1, we designed two siRNAs targeting the back‐splice junction. Compared with the negative control siRNA, si‐circPLCE1#2 provided a more effective knockdown (Figure 2A). Cell viability was then evaluated using the CCK‐8 assay, which indicated that circPLCE1 knockdown suppressed cell proliferation (Figure 2B). Similarly, silencing of circPLCE1 significantly suppressed the colony formation ability of CRC cells (Figure 2C). Moreover, a Transwell assay was performed to determine the impact of circPLCE1 on CRC cell migration and invasion. circPLCE1 knockdown inhibited CRC cell migration and invasion (Figure 2D, E). Finally, the flow cytometry results showed that circPLCE1 knockdown increased the number of early apoptotic cells (Figure 2F), and inhibited the G1‐ to S‐phase transition of the cell cycle (Figure 2G).

**FIGURE 3:**
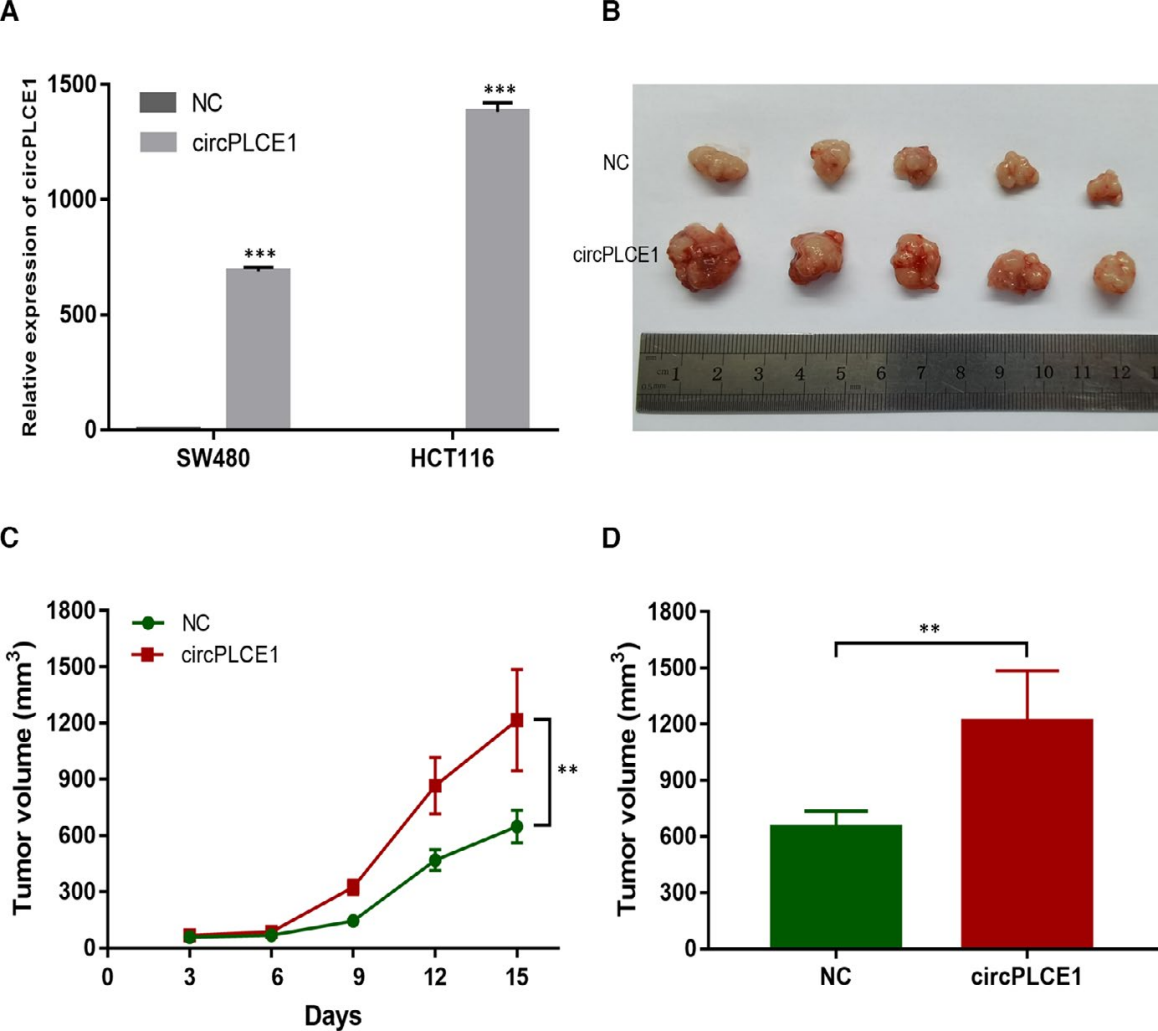
circPLCE1 promotes CRC growth in vivo. (A) Expression of circPLCE1 in SW480 and HCT116 cells after infection with circPLCE1‐overexpression lentiviruses or NC lentiviruses. (B) Xenograft tumours of nude mice 15 days after injection of HCT116 cells (n = 5 per group). (C) Tumour volumes were measured every 3 days. (D) Tumour volumes were measured after the nude mouse was executed. All data are presented as the means ±SD. **P < .01, ***P < .001

To further confirm the biological role of circPLCE1 in the malignant phenotype of CRC cells in vivo, we constructed xenograft tumour models. We developed SW480 or HCT116 cells with circPLCE1 stably overexpressed using pHBLV‐CMV‐circ‐EF1‐Puro‐N lentiviral vectors, and circPLCE1 levels were verified by qRT‐PCR (Figure 3A). Next, HCT116 cells transfected with circPLCE1‐overexpressing vector or negative control vector were subcutaneously injected into BALB/c nude mice (five per group). Our results revealed that ectopic expression of circPLCE1 significantly promoted tumour growth (Figure 3B–D).

### 3.3 PLCE1 mRNA expression was antagonized by circPLCE1

**FIGURE 4:**
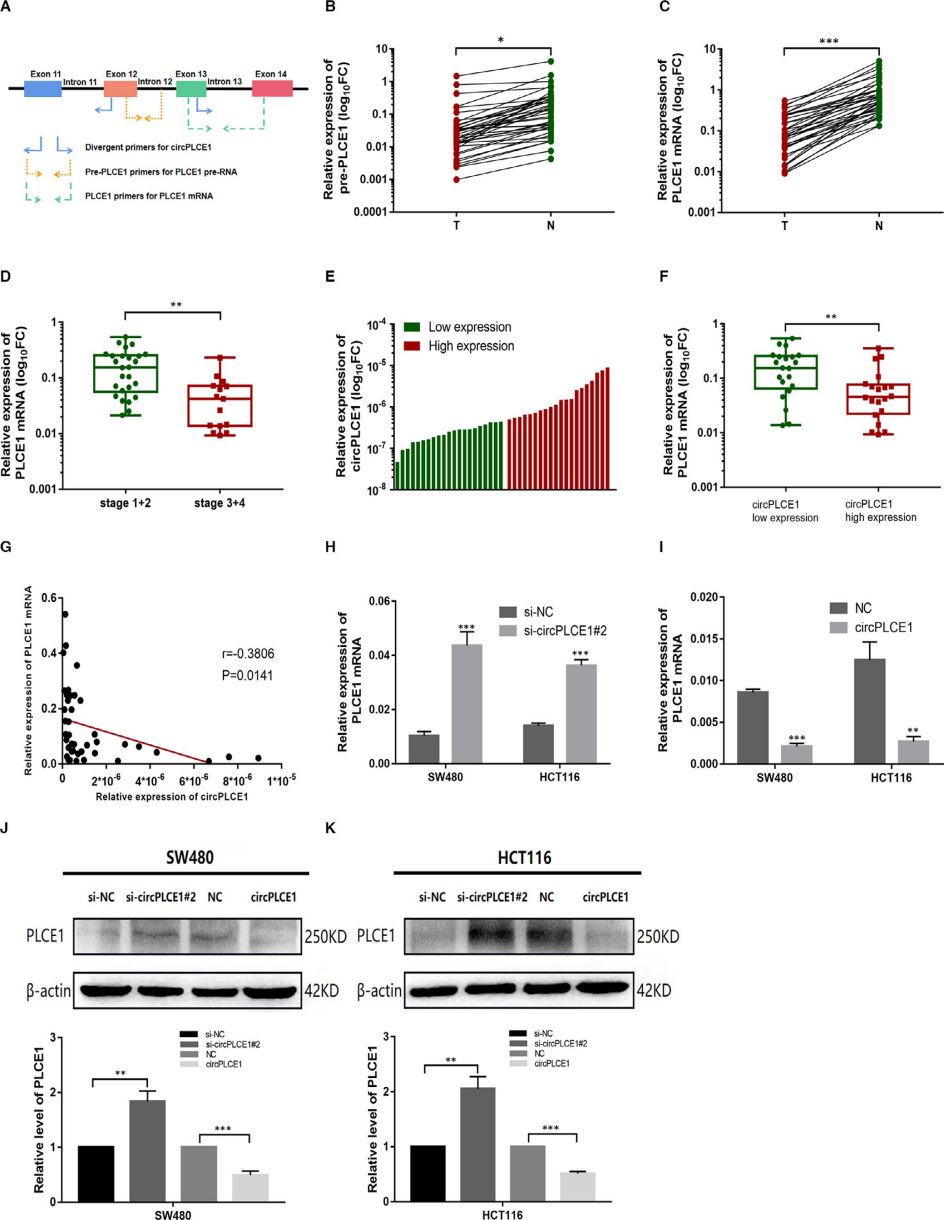
PLCE1 mRNA expression was antagonized by circPLCE1 in CRC tissues and cell lines. (A) Primers to distinguish between circPLCE1, pre‐PLCE1 and PLCE1 mRNA. (B, C) Pre‐PLCE1 and PLCE1 mRNA expressions were significantly decreased in CRC tissues compared with normal tissues. (D) The PLCE1 mRNA expression in patients with different TNM stages. (E) Using the median expression level of circPLCE1 as a cut‐off value, the 41 CRC tissues were divided into low‐ and high‐expression groups. (F) The PLCE1 mRNA expression in low‐ and high‐expression groups. (G) Correlation between circPLCE1 expression and PLCE1 mRNA expression in CRC samples. (H, I) The PLCE1 mRNA expression was detected through qRT‐PCR after overexpression or silencing of circPLCE1 in CRC cells. (J, K) The protein levels of PLCE1 were detected through Western blotting after overexpression or silencing of circPLCE1 in CRC cells. Data are listed as means ±SD. *P < .05, **P < .01, ***P < .001

In the above experiment, we observed an interesting phenomenon in which circPLCE1 was decreased in CRC tissues, but was a tumour promoter. Mechanistically, circPLCE1 was derived from back‐splicing of pre‐PLCE1. The primers for pre‐PLCE1 and PLCE1 mRNA were designed (Figure 4A), and the accuracy of the conjunction was confirmed by Sanger sequencing. Moreover, we experimentally confirmed that pre‐PLCE1 and PLCE1 mRNA expressions were markedly lower in CRC tissues compared with those in paired adjacent normal tissues (Figure 4B, C). Statistical analysis revealed that low pre‐PLCE1 expression levels were not related to clinicopathological features in CRC patients, whereas low PLCE1 mRNA expression was considered a tumour suppressor. As presented in Table 2 and Figure 4D, reduced PLCE1 mRNA expression was related to lymph node metastasis (*P* = .02), distant metastasis (*P* = .03), TNM tumour stage (*P* = .02) and lymphovascular invasion (*P* = .04). Subsequently, we divided the 41 CRC patients into the low‐ and high‐expression groups using the median expression level of circPLCE1 as a cut‐off value (Figure 4E). The results revealed that CRC patients in the high‐expression circPLCE1 group exhibited lower PLCE1 mRNA expression than the CRC patients in the low‐expression circPLCE1 group (Figure 4F). Similarly, we observed a significant negative correlation between circPLCE1 expression and PLCE1 mRNA expression levels (*r* = −.3806, *P* = .0141 for Spearman correlation; Figure 4G).

To further test the association between circPLCE1 and PLCE1 mRNA, we constructed circPLCE1‐silence and circPLCE1‐overexpression cells. The results indicated that PLCE1 mRNA was remarkably increased in response to circPLCE1 inhibition in both SW480 and HCT116 cell lines (Figure 4H). Overexpression of circPLCE1 showed the opposite phenomenon (Figure 4I). However, there was no significant variation in pre‐PLCE1 levels. Western blot analysis was used to detect the expression of PLCE1 protein in CRC cells. The trends of PLCE1 protein were similar to those of mature mRNA of PLCE1, indicating that the process of translation was not influenced (Figure 4J, K). Collectively, these findings indicate that circPLCE1 plays a fundamental role in PLCE1 mRNA processing.

### 3.4 circPLCE1 suppressed RNA splicing of pre‐PLCE1 by binding to SRSF2 protein

**FIGURE 5:**
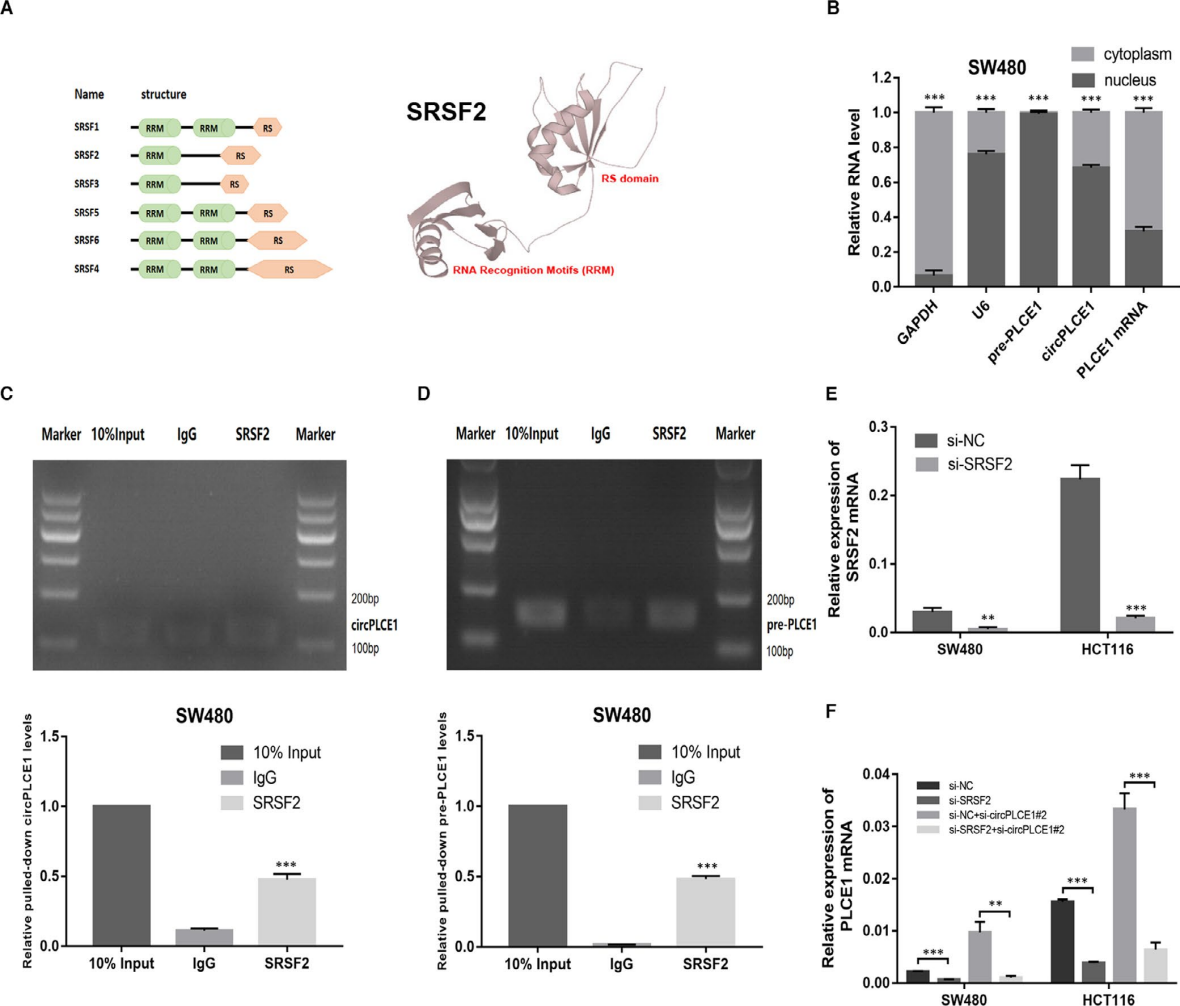
Binding of circPLCE1 with SRSF2 protein. (A) Structure of classic SR proteins, which are characterized by one or two RRMs at the N‐terminus and the signature RS domain at the C‐terminus. (B) Subcellular location of circPLCE1, pre‐PLCE1 and PLCE1 mRNA in SW480 cells. (C, D) RNA immunoprecipitation experiments were performed using anti‐SRSF2 antibody in SW480 cells by qRT‐PCR. Nucleic acid electrophoresis was performed to detection the PCR products. (E) Expression of SRSF2 mRNA in SW480 and HCT116 cells treated with SRSF2 siRNA. (F) Expression of PLCE1 mRNA in SW480 and HCT116 cells treated with SRSF2 siRNA or circPLCE1 siRNA

Next, we deciphered the possible mechanism by which PLCE1 mRNA processing was repressed by circPLCE1. We used the catRAPID website to predict the potential protein interactions with circPLCE1 (Table 3). Among these candidates, SRSF2 was selected as the downstream target of circPLCE1. SRSF2 is the only SR family member that is exclusively localized in the nucleus and is therefore restricted to nuclear processes (Figure 5A). Next, we analysed the interaction strength of SRSF2 with circPLCE1 or pre‐PLCE1 using a computer algorithm (RPISeq). Bioinformatic analysis suggested that circPLCE1 or pre‐PLCE1 had promising potential for binding with SRSF2 protein (Table 4).

**TABLE 3:** RNA‐Protein Interaction Prediction (catRAPID)

RNA input	Protein	Z‐score	Discriminative Power(%)	Interaction Strength(%)
circPLCE1	SRSF9	−0.34	32	80
circPLCE1	SRSF2	−0.37	28	76
circPLCE1	SFPQ	−1.29	10	2

**TABLE 4:** RNA‐Protein Interaction Prediction (RPISeq)

	circPLCE1 / SRSF2	pre‐PLCE1 / SRSF2
Prediction using RF classifier	0.6	0.9
Prediction using SVM classifier	0.86	0.97

Interaction probabilities generated by RPISeq range from 0 to 1. In performance evaluation experiments, predictions with probabilities >0.5 were considered “positive," that is, indicating that the corresponding RNA and protein are likely to interact.

To further verify the molecular mechanisms of circPLCE1 in CRC cells, we first analysed the subcellular distribution of circPLCE1. The results of qRT‐PCR analysis after cell fractionation showed that circPLCE1 was mainly localized in the nucleus (Figure 5B). Next, we conducted RNA immunoprecipitation (RIP) assays and found that circPLCE1 and pre‐PLCE1 were more significantly immunoprecipitated by anti‐SRSF2 antibody compared with negative anti‐IgG antibody (Figure 5C, D). To clarify the function of SRSF2 in PLCE1 mRNA processing, we designed siRNAs for SRSF2 mRNA (Figure 5E). Silencing of SRSF2 decreased the expression of PLCE1 mRNA, and silencing of circPLCE1 could not rescue the SRSF2‐silence–mediated decline of PLCE1 mRNA (Figure 5F).

### 3.5 Identification of the binding sites of circPLCE1 and SRSF2 protein

**FIGURE 6:**
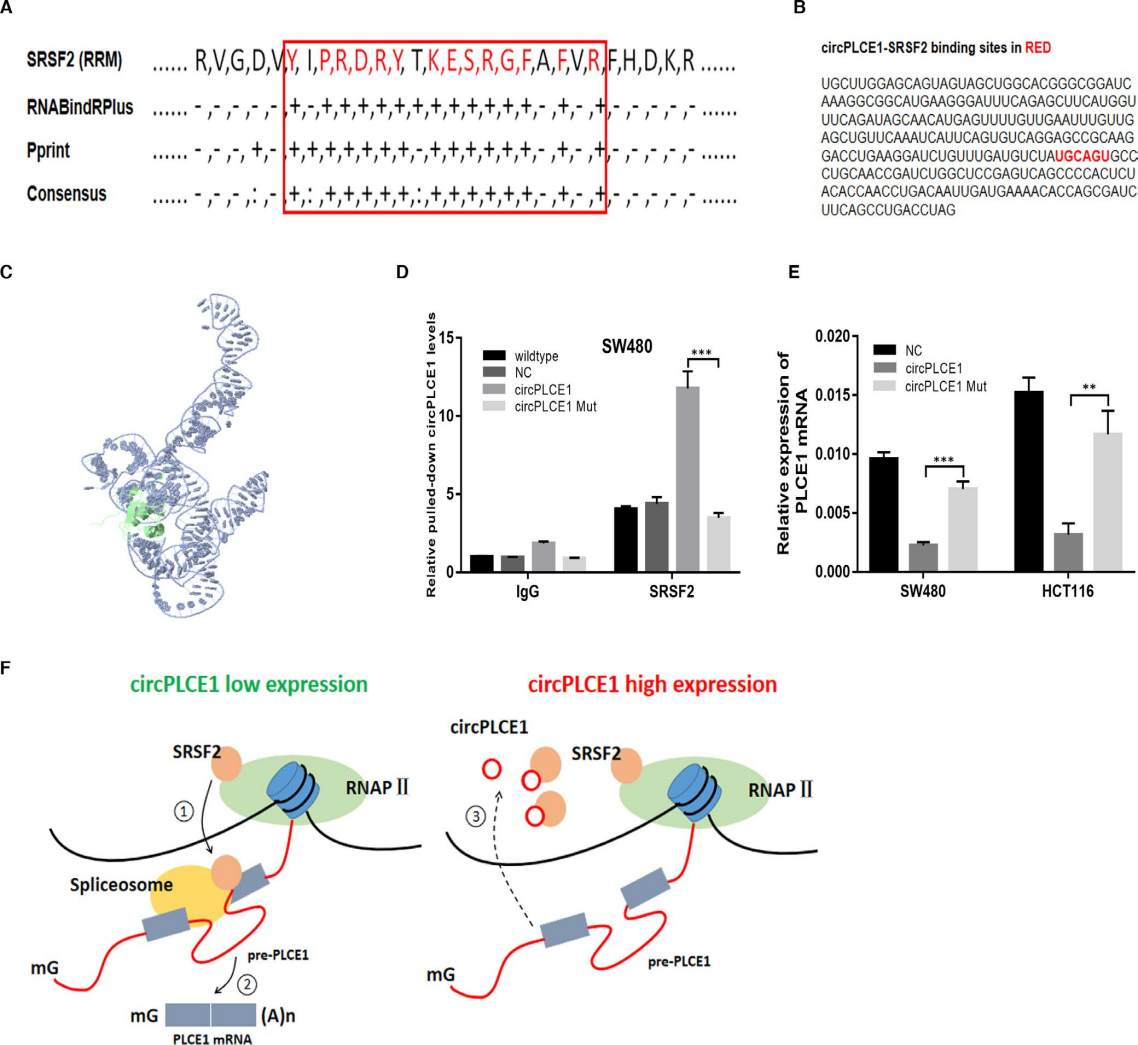
Identify the binding sites of circPLCE1 with SRSF2. (A) Prediction of probable RNA‐binding residues of SRSF2 was carried out by submitting the SRSF2 sequence to Pprint and RNABindRPlus servers. “+” indicates the predicted RNA‐binding residues. (B) The interactive sites of circPLCE1 with SRSF2 were highlighted in red. (C) Graphical representation of three‐dimensional structures of the docking models of circPLCE1 with the binding fragment of SRSF2 RRM by NPDock. (D) In SW480 cells, SRSF2 antibody precipitated higher levels of circPLCE1 in the circPLCE1‐overexpression cells relative to the wild‐type, NC and circPLCE1‐Mut‐overexpression cells. (E) PLCE1 mRNA expression in CRC cells transfected with NC vector, circPLCE1, circPLCE1 Mut was examined by qRT‐PCR. (F) Model depicting the proposed mechanism of suppression of PLCE1 pre‐RNA splicing by circPLCE1. In the circPLCE1 low‐expression CRC cells, SRSF2 is responsible for initiating spliceosome assembly on pre‐PLCE1 ①, which promote the production of PLCE1 mRNA ②. Conversely, in the circPLCE1 high‐expression CRC cells, circPLCE1 is directly binding to SRSF2 and suppress the process of PLCE1 pre‐RNA splicing ③

To identify the binding sites of circPLCE1 with SRSF2, we constructed a docking model using computational approaches. RNABindRPlus and Pprint servers predicted that the RNA‐binding residues were concentrated in the RRM region of the SRSF2 protein (Figure 6A). Previous studies have demonstrated that the SRSF2 RRM region strongly binds to the RNA sequence 5’‐UGCAGU‐3’, which was also found in circPLCE1^13^(Figure 6B). Finally, NPDock was used to perform the in silico molecular docking of circPLCE1 with SRSF2 (Figure 6C).

A plasmid containing the circPLCE1‐SRSF2 binding site mutation (Supplementary Table 3) in circPLCE1 was constructed and transfected into CRC cells. Subsequent RIP experiments showed that the circPLCE1 Mut could not be pulled down by the SRSF2 antibody (Figure 6D). In addition, inhibition of PLCE1 mRNA expression by circPLCE1 was abolished by mutating the binding sites of circPLCE1 with SRSF2 (Figure 6E). The in vitro cell experiments supported the conclusion that circPLCE1 could dock the SRSF2 RRM region.

## 4 DISCUSSION

It has been well‐established that PLCE1 plays a critical role in inhibiting CRC progression.^7^ Exploring distinctive and effective mechanisms of increasing the expression of PLCE1 protein may have better therapeutic efficacy with minimal side effects for CRC treatment. In our previous studies, we analysed the levels of circRNAs in human CRC tissues and paired normal mucosal tissues using high‐throughput sequencing. We found that the novel circRNA circPLCE1 was down‐regulated in cancer samples. Subsequently, 41 paired clinical tissue samples were used to validate the expression of circPLCE1. The results revealed that circPLCE1 expression was exactly decreased in CRC, which was consistent with the previous analytical results from circRNA sequencing. However, clinical data analysis showed that high circPLCE1 expression was significantly correlated with deeper invasion, more lymph node metastasis and advanced TNM stage. These findings indicate that circPLCE1 might act as a tumour promoter in CRC progression.

Similarly, in vitro and in vivo experiments have shown that circPLCE1 plays a vital role as a promoter of CRC progression. Functional assays revealed that knockdown of circPLCE1 restricted cell proliferation and invasion and promoted cell apoptosis to some extent. Moreover, circPLCE1 repression was found to inhibit the G1‐ to S‐phase transition of the cell cycle. We next aimed to elucidate the biological role of circPLCE1 in a xenograft model and found that overexpression of circPLCE1 promoted the growth of CRC cells in BALB/c nude mice. In summary, the opposite functions of circPLCE1 and PLCE1 proteins in CRC suggest that the underlying mechanisms of circPLCE1 require more careful dissection.

By evaluating the expression of pre‐PLCE1, circPLCE1 and PLCE1 mRNA in 41 paired CRC tissues, we discovered that the expression levels of PLCE1 mRNA were negatively correlated with circPLCE1, but showed no correlation with pre‐PLCE1. Moreover, knockdown or overexpression of circPLCE1 in CRC cells could change the levels of PLCE1 mRNA and its translation products. We deciphered the possible molecular mechanism of circPLCE1 by focusing on its ability to compete with SRSF2‐dependent PLCE1 pre‐RNA splicing. SRSF2 is a well‐known member of the SR‐rich family that regulates splicing events.^21^, ^22^ SRSF2 knockdown remarkably reduced PLCE1 mRNA expression. Using bioinformatic methods, we found that circPLCE1 strongly interacted with the SRSF2 protein. We also identified the potential binding sites of circPLCE1 with SRSF2. In this study, by performing RNA fractionation and RIP assays, we confirmed that circPLCE1 was able to bind SRSF2 in the nucleus. Knockdown of circPLCE1 increased PLCE1 mRNA expression in CRC cells, and knockdown of SRSF2 abolished this process. Moreover, we blocked the interplay between circPLCE1 and SRSF2 using a mutant construct targeting the binding sites. Thereafter, the suppression of PLCE1 mRNA production by circPLCE1 overexpression was abolished.

Recently, evidence has accumulated that circRNAs are frequently dysregulated in human cancer and affect tumorigenesis, invasion and metastasis.^23^, ^24^ Many molecular functions are performed by circRNAs to implement their cellular effects, such as interacting with different proteins to form specific RNPs and influence their intrinsic function.^25^, ^26^, ^27^ In our study, we present evidence that PLCE1 mRNA production is negatively controlled by its circRNA via suppression of pre‐PLCE1 splicing. One of our main findings is that circPLCE1 directly binds to the SRSF2 protein and suppresses the spliceosome assembly on nascent pre‐PLCE1 to catalyse intron removal before the completion of transcription (Figure 6F).

The role of circPLCE1 in the genesis and progression of CRC tumours remains contradictory. In our experiments, we found that the expression of circPLCE1 was higher in normal compared with cancerous tissues and in advanced‐stage tumours compared with early‐stage tumours. This phenomenon has indicated that the expression of circPLCE1 may be regulated by some upstream molecules. However, the small sample size limits the generalizability of this study. Moreover, investigations are required to understand circPLCE1 functions in CRC cells both in the nucleus and in the cytoplasm. Although we have discovered that circPLCE1 represses PLCE1 pre‐RNA splicing in the nucleus, the role of circPLCE1 in the cytoplasm remains unknown. Finally, the theoretical basis of this hypothesis is that pre‐PLCE1, SRSF2 protein and circPLCE1 are located in proximity to the nucleus, indicating that the mechanism of action requires visual evidence under a microscope.^28^

In summary, our study uncovered a novel mechanism underlying the direct regulation of PLCE1 mRNA expression through SRSF2‐dependent RNA splicing and provided a molecular basis for a new understanding of the pathophysiological function of circPLCE1. In addition, due to the critical role of PLCE1 in tumour progression, our findings may also pave the way for the pursuit of circPLCE1 as a potential target for CRC treatment.

## CONFLICT OF INTEREST

The authors declare that they have no competing interests.

## AUTHOR CONTRIBUTIONS

**Zhilei Chen:** Methodology (equal); Writing‐original draft (equal); Writing‐review & editing (equal). **Hongyu Chen:** Visualization (equal). **Lei Yang:** Data curation (equal). **Xiangnan Li:** Software (equal). **Zhenjun Wang:** Project administration (equal); Supervision (equal).

## Supporting information

Supinfo

## Data Availability

The data used to support the findings of this study are available from the corresponding author on reasonable request.
