# Supplementary material for: CircPLCE1 facilitates the malignant progression of colorectal cancer by repressing the SRSF2‐dependent PLCE1 pre‐RNA splicing
Source: J Cell Mol Med. 2021 Jun 26;25(15):7244–56. doi: 10.1111/jcmm.16753 (PMC8335689; doi:10.1111/jcmm.16753)
Supplement: Supplementary file 1 — Supinfo [file JCMM-25-7244-s001.docx]

**SUPPLEMENTARY MATERIAL**

**SUPPLEMENTARY TABLE 1** PCR primers

| circPLCE1 forward | 5’-AGCCCCACTCTACACCAACC-3’ |
| --- | --- |
| circPLCE1 reverse | 5’-TTCATGCCGCCTTTGATCCG-3’ |
| pre-PLCE1 forward | 5’-GGATCAAAGGCGGCATGAAG-3’ |
| pre-PLCE1 reverse | 5’-TGAGGAATGGAAAACACCTCTCA-3’ |
| PLCE1 mRNA forward | 5’-CAGCCCCACTCTACACCAAC-3’ |
| PLCE1 mRNA reverse | 5’-GCTCTCAATCCCAGTGCCAT-3’ |
| GAPDH forward | 5’-AATCCCACTACCATCTTCCA-3’ |
| GAPDH reverse | 5’-TGGACTCCACGACGTACTCA-3’ |

**SUPPLEMENTARY TABLE 2** siRNA sequences

| si-circPLCE1#1 | 5’-CAGCCUGACCUAGUGCUUGTT-3’ |
| --- | --- |
| si-circPLCE1#2 | 5’-CUGACCUAGUGCUUGGAGCTT-3’ |
| si-SRSF2 | 5’-GAGCAGGUUUGUCUUUAAATT-3’ |
| negative control | 5’-UUCUCCGAACGUGUCACGUTT-3’ |

**SUPPLEMENTARY TABLE 3** Sequences of circPLCE1 and circPLCE1 Mut

| Sequences of circPLCE1 | UGCUUGGAGCAGUAGUAGCUGGCACGGGCGGAUCAAAGGCGGCAUGAAGGGAUUUCAGAGCUUCAUGGUUUCAGAUAGCAACAUGAGUUUUGUUGAAUUUGUUGAGCUGUUCAAAUCAUUCAGUGUCAGGAGCCGCAAGGACCUGAAGGAUCUGUUUGAUGUCUA**UGCAGU**GCCCUGCAACCGAUCUGGCUCCGAGUCAGCCCCACUCUACACCAACCUGACAAUUGAUGAAAACACCAGCGAUCUUCAGCCUGACCUAG |
| --- | --- |
| Sequences of circPLCE1 Mut | UGCUUGGAGCAGUAGUAGCUGGCACGGGCGGAUCAAAGGCGGCAUGAAGGGAUUUCAGAGCUUCAUGGUUUCAGAUAGCAACAUGAGUUUUGUUGAAUUUGUUGAGCUGUUCAAAUCAUUCAGUGUCAGGAGCCGCAAGGACCUGAAGGAUCUGUUUGAUGUCUA**ACGUCA**GCCCUGCAACCGAUCUGGCUCCGAGUCAGCCCCACUCUACACCAACCUGACAAUUGAUGAAAACACCAGCGAUCUUCAGCCUGACCUAG |
